# Chemical Analysis and Study of Phenolics, Antioxidant Activity, and Antibacterial Effect of the Wood and Bark of *Maclura tinctoria* (L.) D. Don ex Steud.

**DOI:** 10.1155/2012/451039

**Published:** 2012-02-19

**Authors:** K. C. Lamounier, L. C. S. Cunha, S. A. L. de Morais, F. J. T. de Aquino, R. Chang, E. A. do Nascimento, M. G. M. de Souza, C. H. G. Martins, W. R. Cunha

**Affiliations:** ^1^Instituto de Química, Universidade Federal de Uberlândia, Avenida João Naves de Ávila 2121, 38400-902 Uberlândia, MG, Brazil; ^2^Núcleo de Pesquisas em Ciências Exatas e Tecnológicas, Universidade de Franca, Avenida Dr. Armando Salles Oliveira 201, 14404-600 Franca, SP, Brazil

## Abstract

*Maclura tinctoria* (L.) D. Don ex Steud. has one of the highest qualities among the coefficients for Brazilian woods (up to 9.6) and resistance rates equivalent to Indian teak (*Tectona grandis*). In this study, the macromolecular constituents and total phenols compounds as well as the antioxidant and antibacterial activities of this wood were evaluated. Total phenols and proanthocyanidin levels were higher in wood when compared with bark levels. The antioxidant activity of wood extracts (IC_50_ = 18.7 **μ**g/mL) was more effective than that of bark extracts (IC_50_ = 20.9 **μ**g/mL). Wood and bark extracts revealed a high potential for inhibition of aerobic and anaerobic bacteria. The bark extracts were the most active (MIC from 20 to 60 **μ**g/mL). Both antioxidant activity and high potential for bacteria inhibition turn these extracts promising for drug formulations, especially as antibacterial agent.

## 1. Introduction

Since the beginnings of civilization, humans have used natural products for healing the ills that afflict them. Plants are biochemical labs that produce inside their cells a variety of complex substances with numerous active compounds. With the advent of the pharmaceutical chemistry at the beginning of the nineteenth century, plants became the primary source of substances for drug development [1–3].

The Moraceae family, which is predominantly tropical, has around 63 genera and 1500 species. In Brazil, studies have indicated the existence of 28 genera with about 340 species represented by trees, shrubs, grasses, vines, and even epiphytes [4]. The genus *Maclura* consists of eleven species with an exclusively tropical distribution. Three species occur in the Americas from the United States to Argentina, and in Brazil only *Maclura tinctoria* and *Maclura brasiliensis* occur [5].

*Maclura tinctoria *(L.) D. Don ex Steud. (botanical synonymy: *Chlorophora tinctoria *(L.) Gaud.) is an arboreal, dioecious, thorny, milky, and semideciduous species, usually 10 to 20 meters high and 40 to 60 cm in diameter [5]. It is known as “amoreira,” “amarelinho,” or “taiúva,” and this species presents a moderately heavy wood, which is flexible and very durable even under the most adverse conditions and is highly resistant to xylophagous organisms under conditions favorable to decay. It is used in furniture making, carpentry, and external constructions, such as in posts, pillars, and fences [6, 7]. According to Gonzaga [8], this wood is recommended for naval construction and it is considered to be wasted when used for sleepers, piles, poles, cross arms and rustic external works. Also according to Gonzaga [8], the wood of *Maclura tinctoria* has one of the highest qualities among the coefficients for Brazilian woods (up to 9.6) and resistance rates equivalent to Indian teak (*Tectona grandis*). There are no scientific results regarding its durability, a more important feature than strength and hardness. However, even in contact with soil, moisture, and climate adversity, it is popularly said that this species has a lifetime warranty and that this wood can be found well preserved as pillars or house joists with more than 200 years old.

In folk medicine, the stem exudate and bark tea are used by the native population for their anti-inflammatory properties. The resin is used as a dye and against toothache. Its fruits are edible and have a very nice, sweet flavor and can be consumed raw or in juices and pastries [9, 10].

The only studies found in the literature involving chemical analysis of *Maclura tinctoria* species refer to the isolation and identification of flavonoids, xanthones, flavones, and chalcone glycosides from bark extracts from the forest regions of Bolivia, Peru, or Venezuela [10–13]. Some of the compounds identified presented anti-HIV [13], antifungal [12], and antioxidant activity equivalent to trolox and *β*-carotene [10].

There are no studies regarding the chemical composition of the wood of this species, so the main objective of this work is to quantify and characterize the macromolecular constituents, total phenolics content, and the antibacterial and antioxidant activity of the wood and bark of *Maclura tinctoria*.

## 2. Materials and Methods

### 2.1. Solvents, Reagents, and Solutions

Analytical grade solvents and reagents were used. The Folin-Ciocalteau reagent was acquired from Merck, Brazil. The DPPH free radical (2,2-diphenyl-1-picrylhydrazyl) and catechin, gallic acid, butylhydroxytoluene (BHT), and ascorbic acid standards were from Sigma Chemical Co. All experiments were performed in triplicate, and the results correspond to the mean ± the standard deviation.

### 2.2. Origin and Preparation of Wood and Bark Samples

Wood and bark samples of *Maclura tinctoria* were collected in a nature reserve located in the city of Perdões (latitude 21° 02′06.61′′S, longitude 45° 0.7′11.91′′W, altitude of 913 m) in Minas Gerais State, Brazil. The samples were obtained in three distinct regions, where the tree occurs naturally, in a transition area between Cerrado and the Atlantic Forest. This species (voucher) was identified by Professor Dr. Julio Cesar Viglione Penna from the Institute of Agricultural Sciences from the Federal University of Uberlandia.

 Three specimens of about 8 years old and 15 cm in diameter were cut in February 2007 at chest height (0.7 to 1.3 meters). The wood was cut into logs and dried in ambient room air for 120 days. The wood was debarked and cut into 2 cm discs, ground in a ball mill, and sifted through a 40–80 mesh steel sieve. The same grinding process was done with the bark. The moisture content in the wood and bark was determined and used in calculations when needed.

### 2.3. Macromolecular Constituents

#### 2.3.1. Extractives

Extractives determination was performed at the same time as the extractive-free wood was prepared for macromolecular constituent quantification and followed the TAPPI T264 om-88 method, using cyclohexane instead of benzene. The solvent sequences employed were cyclohexane : ethanol (2 : 1, v/v), ethanol (95%), and water, respectively. The extractives were dried in an oven at 105 ± 2°C until a consistent weight was achieved.

 The determination of the macromolecular composition of the bark had some differences to that performed on the wood, since applying the same methodology for wood and bark could lead to incorrect conclusions [14]. According to Browning [14], the bark has a more variable composition than wood, and a prior chemical treatment with NaOH is needed before any analysis. The alkali treatment of the bark was performed according to the TAPPI T212 om-93 method.

#### 2.3.2. Soluble and Insoluble Lignin

The level of insoluble lignin in sulfuric acid in the wood and bark was performed according to TAPPI T222 om-88 and the soluble lignin level was determined by spectrophotometry, according to the method described by Goldschmidt [15].

#### 2.3.3. Holocellulose, *α*-Cellulose, and Hemicellulose

The wood and bark holocellulose levels were determined by the method described by Browning [14]. The *α*-cellulose percentage was quantified from the holocellulose according to the TAPPI T203 om-83 method. The hemicellulose level was determined by the difference between the holocellulose and *α*-cellulose levels [16].

### 2.4. Determination of Total Phenols Content

The raw extracts of wood and bark were prepared by extraction using methanol : water (4 : 1, v/v) and acetone : water (7 : 3, v/v), as recommended by Hagerman [17].

 The total phenols levels were determined from raw extracts of wood and bark by Folin-Ciocalteau [18–20]. The results were expressed as gallic acid equivalents (mg of GAE/g of wood or bark).

#### 2.4.1. Determination of Proanthocyanidin

Proanthocyanidin was determined by the vanillin method [21, 22]. The results were expressed as catechin equivalents (mg of CE/g of wood or bark).

### 2.5. Determination of Antioxidant Activity and IC_50_

Antioxidant activity was determined using the stable free radical 2,2-diphenyl-1-picrylhydrazyl (DPPH) following the method described by Brand-Williams et al. [23] and modified by Yildirim et al. [24]. The average effective concentration (IC_50_), which represents the concentration of a sample necessary to sequester 50% of the DPPH radicals, was obtained by plotting the percentage of DPPH-H against the concentration of the extracts in each sample [22, 25].

### 2.6. Antibacterial Activity

The minimum inhibitory concentration (MIC, the lowest concentration of the extract able to inhibit microbial growth) was determined in triplicate by using the broth microdilution method in 96-well microplates [26]. The following standard strains from the ATCC were used: *Streptococcus sanguinis* (ATCC 10556), *Streptococcus mutans* (ATCC 25175), and *Streptococcus mitis* (ATCC 49456)—aerobic bacteria—*Prevotella nigrescens* (ATCC 33563), *Actinomyces naeslundii* (ATCC 19039), and *Porphyromonas gingivalis* (ATCC 33277)—anaerobic bacteria. The inoculums was adjusted for each organism to yield cell concentration of 5 × 10^5^ colony-forming units (CFU/mL), according to previous standardization by the Clinical Laboratory Standards Institute (CLSI) for aerobic bacteria (CLSI, 2006) and 1 × 10^6^ CFU/mL for anaerobic bacteria (CLSI, 2007). Chlorhexidine dihydrochloride was used as positive control.

## 3. Results and Discussions

### 3.1. Wood and Bark Chemical Analysis

#### 3.1.1. Extractives

Table 1 shows the extractive levels found by sequential extraction of wood and bark of *Maclura tinctoria*. The sum of cyclohexane : ethanol and hot water was higher in bark when compared to wood, and only the wood ethanol extract was higher when compared to bark. Usually, barks have a higher level of extractives when compared to wood from the same tree [27, 28].

The high extractive levels and composition of these extractives can contribute to hardness and resistance of *Maclura tinctoria* wood to mechanical attacks and xylophagous organisms even under conditions that would favor decay. Studies made by Findlay and Paes suggest that the resistance to biodegradation is mainly attributed to the presence in the wood of certain substances such as tannins or other complex phenolic substances, the decomposition of which is very difficult for xylophagous organisms [29, 30]. 

Needless to say, extractive level does not completely explain wood resistance. For example, *Eucalyptus *wood has high extractive levels, but it is easily degraded [31]. Besides the extractives, the variation in the amount of cellulose, hemicellulose composition, and the type of lignin and the association of these components will result in a three-dimensional structure of cell walls with specific characteristics for each species of wood and can explain the tensile strength in a more satisfactory way. Compression and moisture can also contribute to defense against insects and pathogens [12]. 

### 3.2. Macromolecular Composition

Table 1 also shows the composition of the macromolecular constituents for the wood of *Maclura tinctoria*. We concluded that *Maclura tinctoria *fits the profile of a hardwood tree, after comparing the lignin content [32]. This wood has higher *α*-cellulose content than hardwood species *Astronium urundeuva* [33] and *Moquinia polymorpha* wood [34], but lower when compared to softwood species *Cedrela fissilis* [35] and *Pinus oocarpa *wood [36] and the hardwood species *Eucalyptus grandis* [31]. The lignin content was in the range expected for hardwoods [32] but less than that of *Astronium urundeuva, Cedrela fissilis, Eucalyptus grandis,* and *Pinus oocarpa*. Therefore, *Maclura tinctoria* and *Astronium urundeuva,* even considered as hardwoods for botanical aspect, have relatively low levels of lignin, which suggests that the amount of lignin is not indicative of the resistance shown by these species. 

Table 1 also presents the average macromolecular composition of *Maclura tinctoria* bark. These studies were compared with barks studied by Kofujita et al. [37]. The holocellulose content in the bark of *Maclura tinctoria* was higher than that in the species studied by Kofujita et al. [37]. The content of soluble and insoluble lignin in the bark of *Maclura tinctoria* was lower [37]. The extractives content was about the same found for the other investigated species. At present, there are no studies in literature regarding the wood and bark at the same time of the same species studied. 

### 3.3. Total Phenols and Proanthocyanidin Analysis

The quantification for total phenols and proanthocyanidin contents in the wood and bark of *Maclura tinctoria* are shown in Table 1. 

The extractions, as recommended by Hagerman, made with acetone : water yielded better results than methanol : water extractions [17]. The yield for acetone : water (7 : 3, v/v) for wood and bark was 20.5 ± 0.1 and 8.4 ± 0.6, respectively, and for methanol : water (4 : 1, v/v) for wood and bark was 14.4 ± 0.6 and 6.6 ± 0.8, respectively. The values were higher than those described for *Eucalyptus* wood (*E. camaldulensis, E. globulus, E. rudis, E. grandis,* and* E. urophila*), using methanol : water as the extraction solvent. These values were in the range of 2.6 to 12.0% [38–40]. The described values found for *Astronium urundeuva* wood were in the range of 18.7 to 22.4% for methanol : water and acetone : water extracts, respectively [33]. The high extractive level in the wood compared to the bark of *Maclura tinctoria* could contribute to explain the high resistance of this wood against xylophagous organisms, even under favorable decay conditions. 

The total phenols level in the assayed extracts was higher for wood when compared to bark, although these results were similar, and the differences when acetone : water was used as an extraction solvent were not significant (*P* > 0.05). Usually, extractive levels in bark are higher than in wood, and similarly, the total phenols level in bark has been shown to be higher [27, 41], although some studies have reported higher polyphenols levels in wood than in bark [42, 43]. 

The values found in the literature for the total phenols content of methanol : water extracts of *Eucalyptus* wood (*E. camaldulensis, E. globulus, *and* E. rudis*) were in the range of 5.9 to 17.5 mg/g, and the values for the bark were in the range of 2.5 and 91.6 mg/g [38–40]. Vázquez et al. reported a value of 14.8 mg/g for *Eucalyptus globulus* bark extracted with this solvent [44]. There is a large influence of the solvent, as shown with *Acacia auriculiformis* bark, which had a yield of 10.9 mg/g with methanol extraction and 300.0 mg/g for acetone extraction [45, 46]. 

The values reported for *Astronium urundeuva *wood were 43.8 mg/g for the acetone : water extract and 37.7 mg/g for the methanol : water extract [19]. So, the values found for *Maclura tinctoria* wood are close to the values reported for *Astronium urundeuva* wood and higher than those reported for many species of *Eucalyptus*. These values are similar to those normally found in vegetable peelings. Therefore, the high phenolics content found in *Maclura tinctoria* and *Astronium urundeuva* wood, in association with the macromolecular composition (other extractives, holocellulose and lignin), can contribute to the high resistance of these woods. 

The wood of *Maclura tinctoria* showed a higher yield for proanthocyanidin when compared to the bark, in the two extracts tested (Table 1). These results were close to those found by acetone : water extraction of *Astronium urundeuva* wood (6.1 mg/g), but much lower when compared to methanol : water (31.2 mg/g) extracts [19]. The values found for methanol : water extracts of *Eucalyptus* wood under the same conditions were in the range of 0.7 and 6.3 mg/g [40]. So, the proanthocyanidin content of *Maclura tinctoria* wood is very much lower when compared to the methanol : water extract of *Astronium urundeuva* wood and to the yield of the acetone : water extract of *Eucalyptus* wood [19, 40]. The comparison of these results suggests that the proanthocyanidin levels probably do not contribute to the high resistance of the wood, as these values are similar to those in the literature for others species that do not show this durability, like *Eucalyptus*. 

### 3.4. Antioxidant Activity

One of the most commonly used methods to determine antioxidant activity is quantification of a free radical scavenging assay, using a molecule such as DPPH [23–25]. 

Both methanol extracts (wood and bark) of *Maclura tinctoria* showed antioxidant activity, but the wood presented a better result. The methanol extracts had fast kinetics, consuming more than 50% of the DPPH radical in the first 5 minutes of the reaction for samples with a concentration nearly 25.0 *μ*g/mL, under the conditions used. The value for the average effective concentration (IC_50_) is shown in Table 1. 

As DPPH consumption increased, the IC_50_ value became lower, and so the antioxidant activity would be higher. Thus, the wood of *Maclura tinctoria* had a higher antioxidant activity than the bark. Several studies indicate that this activity is directly related to the content of phenolic compounds [42, 43], which agrees with the highest concentration of phenolics in wood extracts of *Maclura tinctoria*. 

Comparing the results obtained with others cited in the literature [43, 47–52], we observed that both wood and bark extracts of *Maclura tinctoria* showed IC_50_ values in the range usually found for wood and bark of other species. Comparing these values with well-known antioxidant standards like gallic acid (2.8 *μ*g/mL), catechin (5.5 *μ*g/mL), butylhydroxytoluene (BHT) (17.0 *μ*g/mL), and ascorbic acid (9.4 *μ*g/mL), considered to have excellent antioxidant activity, the wood and bark of *Maclura tinctoria *showed good results, which could allow them to be used as addictives in food, drugs, or other industrial products similarly to what was reported by Brighente et al. in their studies [52]. 

### 3.5. Antibacterial Activity

The wood and bark extracts of *Maclura tinctoria* were tested to verify their antibacterial activity by the broth microdilution method (BMD). The values for minimum inhibitory concentration (MICs) are shown in Table 2. 

The wood and bark extracts were active against *Streptococcus sanguinis *(ATCC 10556), *Streptococcus mitis *(ATCC 49456), and *Streptococcus mutans *(ATCC 25175) at concentrations in the range of 80 to 400 *μ*g/mL. The methanol : water extract of the bark showed an MIC of 80 *μ*g/mL against the major etiologic agent of dental caries. Many authors have reported that brute extracts from natural products with an MIC below 1000 *μ*g/mL are considered relevant and extracts with an MIC below 100 *μ*g/mL are considered promising as potential antimicrobial agents [53, 54]. The cyclohexane : ethanol extract from the bark inhibited the growth of all anaerobic bacteria and showed some of the highest antibacterial activity with MIC of 20 *μ*g/mL for *Prevotella nigrescens* (ATCC 33563) and 60 *μ*g/mL for *Actinomyces naeslundii* (ATCC 19039) and *Porphyromonas gingivalis* (ATCC 33277). 

Herein, all the bacteria tested in the antibacterial assay belonged to strains of the collection (ATCC), which is a shortcoming of this work. However, our research group has published various articles [26, 55–59] that included collection strains only since we consider that these strains are more stable from a genetic viewpoint and would thus represent the bacterium species, thereby enabling comparison with other investigations. The in vitro assay furnishes a reliable indication of how the microorganism is able to respond to the target agent, and extrapolation of the results for that species or even genus should be accepted [60]. Nevertheless, new antibacterial studies must be carried out, in order to include examination of strains isolated from patients. 

 Thus, the extracts are potential antiplaque agents for the prevention of dental caries and more complex lesions such as periodontitis (loss of fibers and bone surrounding the tooth) and endodontic infections (infection of the canal of the tooth). 

## 4. Conclusions

The results of this study show that *Maclura tinctoria* extracts contain a considerable amount of phenolic compounds, and have significant antioxidant activity and antibacterial activity. The extracts for this species can be considered as promising candidates for formulations of antioxidant supplements from natural sources. The raw extracts of the wood and bark exhibited antibacterial effects against Gram-positive and Gram-negative bacteria with higher activity against anaerobic bacteria, especially in the raw cyclohexane : ethanol extract. The results indicated that the bark and wood of *Maclura tinctoria* have potential antibacterial activity, which is very promising for the prevention of dental caries and other oral pathologies. It is supposed that the results of this study will contribute to the recent increase in research with natural products in many areas such as food industries, medicine, pharmaceutical and cosmetic industries, and odontology. Future studies should be carried out to evaluate the in vivo potential of these extracts and isolation and identification of the bioactive compounds in wood and bark extracts of *Maclura tinctoria*. It is suggested that new antibacterial studies must be carried out in order to include examination of strains isolated from patients and further investigations may be accomplished, including evaluation of antifungal activity. 

## Figures and Tables

**Table 1:** Chemical properties for *Maclura tinctoria* wood and bark (% of dry wood/bark).

Chemical properties		Wood	Bark
Solvents extraction (%)	Cyclohexane : ethanol (1 : 2, v/v)	2.8 ± 0.4	20.8 ± 0.2
	Ethanol (95%)	3.9 ± 0.7	1.4 ± 0.7
	Hot water	4.2 ± 0.9	12.5 ± 1.5
	NaOH 1% (m/v)	—	15.9 ± 0.8
	TOTAL	10.8 ± 2.0	50.6 ± 3.5

Macromolecular composition (%)	Total extractives	10.8 ± 1.0	50.6 ± 2.0
	Soluble lignin	1.3 ± 0.00	0.4 ± 0.0
	Insoluble lignin	21.5 ± 1.3	18.8 ± 1.2
	Holocellulose	69.4 ± 2.2	29.1 ± 1.9
	*α*-Cellulose	46.0 ± 1.5	—
	Hemicellulose	23.6 ± 0.9	—
	TOTAL	103.0 ± 4.5	99.0 ± 5.1

Total phenol (mg of GAE/g)	Methanol : water extract (4 : 1, v/v)	38.4 ± 0.4	35.0 ± 0.9
	Acetone : water extract (7 : 3, v/v)	45.3 ± 0.9	43.2 ± 1.2
Proanthocyanidin (mg of CE/g)	Methanol : water extract (4 : 1, v/v)	5.1 ± 0.6	3.9 ± 0.1
	Acetone : water extract (7 : 3, v/v)	6.5 ± 0.6	4.9 ± 0.1

IC_50_ (*μ*g/mL)		18.7 ± 0.5	20.9 ± 0.6

**Table 2:** Values of minimal inhibitory concentration for the extracts of *Maclura tinctoria* against aerobic and anaerobic bacteria in *μ*g/mL.

Extracts	Aerobic bacteria						Anaerobic bacteria					
	Wood			Bark			Wood			Bark		
	*S. sanguinis*	*S. mutans*	*S. mitis*	*S. sanguinis*	*S. mutans*	*S. mitis*	*P. nigrescens*	*A. naeslundii*	*P. gingivalis*	*P. nigrescens*	*A. naeslundii*	*P. gingivalis*
Cyclohexane : ethanol (1 : 2. v/v)	250	250	*	400	*	200	300	200	200	≤ 20	60	60
Ethanol (95%)	400	400	*	*	*	300	300	400	200	200	200	90
Hot water	400	400	*	400	*	400	300	*	300	300	400	300
Methanol : water (4 : 1. v/v)	400	400	*	*	80	*	400	*	300	200	*	300
Acetone : water (7 : 3. v/v)	300	350	*	*	*	400	300	400	200	200	*	100

Control (+)	3.68	0.92	3.68	3.68	0.92	3.68	0.92	0.92	3.68	0.92	1.84	3.68

*Inactive in the tested concentrations (MIC higher than 400 *μ*g/mL); compared to the positive control (chlorhexidine digluconate), the negative control (5% DMSO) did not affect microorganism growth.
